# Accessory pathway properties are similar in symptomatic and asymptomatic preexcitation

**DOI:** 10.1007/s10840-022-01252-7

**Published:** 2022-05-26

**Authors:** Anette Jemtrén, Lennart Bergfeldt, Per Insulander, Aigars Rubulis, Jari Tapanainen, Mats Jensen-Urstad

**Affiliations:** 1 Department of Cardiology, Karolinska Institutet at Karolinska University Hospital Huddinge, Stockholm, Sweden; 2 Department of Cardiology, Sahlgrenska University Hospital, Gothenburg, Sweden; 3 Department of Molecular and Clinical Medicine/Cardiology, Institute of Medicine, Sahlgrenska Academy, University of Gothenburg, Gothenburg, Sweden

**Keywords:** Pre-excitation, Accessory pathways, Electrophysiological study, Sudden cardiac death

## Abstract

**Purpose:**

Patients with WPW syndrome have an increased mortality rate compared to the general population. Although asymptomatic preexcitation has previously been considered benign, recent studies have found that also asymptomatic patients have clinical and electrophysiological factors associated with increased risk of sudden cardiac death. This study compares the baseline electrophysiological characteristics of accessory pathways in symptomatic and asymptomatic patients with preexcitation. We hypothesized that a significant proportion of asymptomatic patients has inducible orthodromic tachycardia during programmed electrical stimulation.

**Methods:**

This retrospective study includes 1853 patients with preexcitation who underwent invasive electrophysiological testing in two Swedish University Hospitals between 1991 and 2018. The mean age was 36 ± 17 years with a range of 3–89 years. Thirty-nine percent was women. A total of 269 patients (15%) were children younger than 18 years. Electrophysiological data included effective refractory period of the accessory pathway (APERP, in 1069 patients), tachycardia cycle length, inducibility and type of tachycardia, and AP localization.

**Results:**

A total of 1703 (93%) patients reported symptoms suggesting tachyarrhythmias before the study and 128 (7%) were asymptomatic. The proportion of potentially dangerous pathways with short APERP (≤ 250 ms) were similar in symptomatic and asymptomatic patients (187/949, 20% vs. 25/108, 23%) (*P* = 0.40) as was the mean APERP (303 ± 68 ms vs. 307 ± 75) (*P* = 0.61). The proportion of patients who had inducible arrhythmia was larger in the symptomatic group (64% vs. 31%) (*P* < 0.001).

**Conclusion:**

The results of this study strengthen the present guideline recommendation (IIA) to consider invasive risk assessment in patients with asymptomatic preexcitation.

## Introduction

Patients with preexcitation and tachycardia related symptoms — i.e. the WPW syndrome — have an increased mortality rate compared to the general population with an incidence of sudden death of about 0.15%/year or 3–4% per lifetime [1–3]; the risk in patients with asymptomatic preexcitation is much less known. In WPW patients, electrophysiological parameters associated with increased risk of potentially life threatening arrhythmias are multiple pathways, inducible arrhythmia, and short AP refractory period [4–6], with limited predictive value associated with each of these parameters [4–6]*.* Recent studies that include both symptomatic and asymptomatic patients have found that even asymptomatic patients may have electrophysiological factors associated with increased risk of potentially life threatening arrhythmias [7, 8]. Most however develop symptoms prior to presenting with life threatening arrhythmias with potential to intervene earlier [7, 8]. Still, the present guidelines have a “may be considered” (IIA) recommendation for invasive electrophysiological study (EPS) for risk evaluation in asymptomatic preexcitation [9].

While patients with the WPW syndrome have been referred for invasive EPS and ablation on a routine basis for > 25 years, this has not been the case for patients with asymptomatic preexcitation. The positive predictive value of an EPS regarding a potentially dangerous AP and the risk for serious adverse events have been considered too low to justify routine EPS and ablation in asymptomatic patients other than those with some high-risk occupations [10]. This strategy, however, was based on small studies. The strategy at our hospitals has been to offer EPS with possible ablation also to asymptomatic patients referred to us [11].

This retrospective observational two-centre study compares the electrophysiological characteristics of APs in symptomatic and asymptomatic patients with preexcitation. We hypothesized that the electrophysiological properties of APs would be similar in symptomatic and asymptomatic (or not yet symptomatic) patients and that a significant proportion of asymptomatic patients would have inducible tachycardia suggestive of latent WPW syndrome.

## Methods

### Ethical approval

Approval was obtained from the local Ethics committee in Stockholm, DN:2016/1481–31/4 (2019–01,205).

### Study population

We identified 1853 patients with pre-excitation who underwent their first invasive EPS at Karolinska University Hospital in Stockholm, Sweden, and Sahlgrenska University Hospital in Gothenburg, Sweden, between 1991 and 2018. Baseline characteristics are shown in Table 1.

**Table 1:** Baseline patient characteristics

	**Asymptomatic patients** *N* = 128	**Symptomatic patients** *N* = 1703	*p*
**Variable**			
**Age(years)**	32.7 ± 13.3	36.3 ± 16.7	0.02
**Gender, female**	39/128 (30.4%)	89/1703 (39.9%)	0.04
**Underlying heart disease**			
**Yes**			
Hypertension Ischemic heart disease Heart failure Valve disease Congenital heart disease Cardiomyopathy	4 (3.1%) 1 (0.8%) 2 (1.6%) 1 (0.8%)	178 (9.8%) 77 (4.2%) 39 (2.2%) 9 (0.6%) 9 (0.5%) 46 (2.5%) 6 (0.3%) 9 (0.5%)	0.007
**Symptoms**			
Palpitations Presyncope or syncope Sudden cardiac arrest	n/a n/a n/a	1622 (95.2%) 533 (31.3%) 5 (0.3%)	

### Analysed data

Medical records were assessed regarding previous heart disease, symptoms correlated to arrhythmia, ECG documentation of arrhythmia, and adverse events related to the invasive procedure. Data from the EPS were collected from the local EP registries. Electrophysiological data included effective refractory period of the AP (APERP), tachycardia cycle length (TCL), shortest preexcited R-R interval (SPRRI) during AF, inducibility and type of tachycardia, and AP localization. The APERP was defined by programmed electrical stimulation (PES) applying the extra-stimulus technique (S1S2) usually at a S1 cycle length of 600 or 500 ms with a shortest S1S2 of 200 ms.

### Statistical analysis

Numerical variables are expressed as mean ± standard deviation. Continuous variables were compared using the Student’s *t*-test and categorical variables were compared using the chi-squared test or Fisher’s exact test. A *P* < 0.05 was considered statistically significant. Statistical analyses were performed using SPSS version 25 software (IBM SPSS Inc., Chicago, IL, USA).

## Results

### Overall results

The mean age at the time for the EP study was 36 ± 17 years with a range of 3–89 years. In all, 269 patients (15%) were children younger than18 years. At the time of the procedure, women were older (37 ± 17 years) than men (35 ± 16 years) (*P* = 0.04). On average, the symptomatic group was 3 years older than the asymptomatic group (*P* = 0.02), and the proportion of men in the asymptomatic group (69.5%) was higher than in the symptomatic group (60.1%) (*P* = 0.04) but did not differ in any other aspect (Table 1). In all, 182 (9.8%) patients had a concomitant cardiovascular disease, most commonly hypertension (4.2%), congenital heart disease (2.5%), or ischemic heart disease (2.2%). The average age in the group with underlying cardiovascular disease (50 ± 19) was higher than the group without (35 ± 16 years) (*P* = 0.02) (Table 1).

**Fig. 1:**
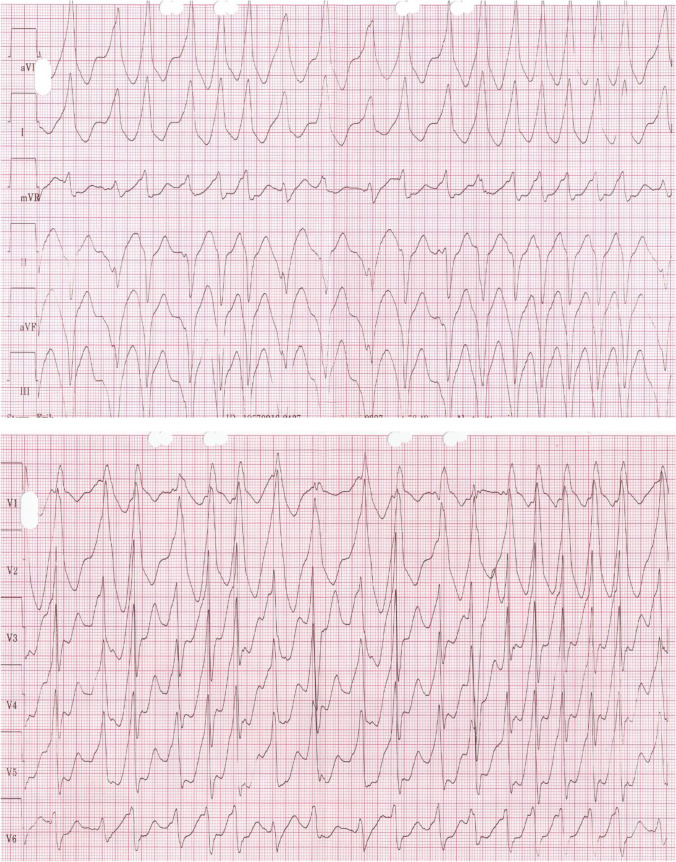
Preexcited atrial fibrillation. Shortest R-R interval 200 ms (paper speed 50 mm/s)

The majority, 1703 patients (93%), reported symptoms suggesting tachyarrhythmias before the EPS, and 128 (7%) patients were asymptomatic (Table 1). Data regarding symptoms were missing in 32 patients. The most frequent symptom was palpitations, which was reported by 1622/1703 (95.2%) patients. More severe symptoms such as pre-syncope and syncope were reported by 533/1703 patients (31.3%). Five patients (0.3%) had experienced sudden cardiac arrest. One or more tachycardia was documented in 948 (51%) patients before the procedure. Orthodromic atrioventricular reciprocating tachycardia (AVRT), the most common tachycardia, was found in 880 (68%) of them. Antedromic tachycardia was found in 138 patients (15%), atrial fibrillation in 230 patients (23%) and preexcited atrial fibrillation in 244 (24%) of the patients (Fig. 1). Ventricular tachycardia (VT) or ventricular fibrillation (VF) was documented in 14 patients.

**Fig. 2:**
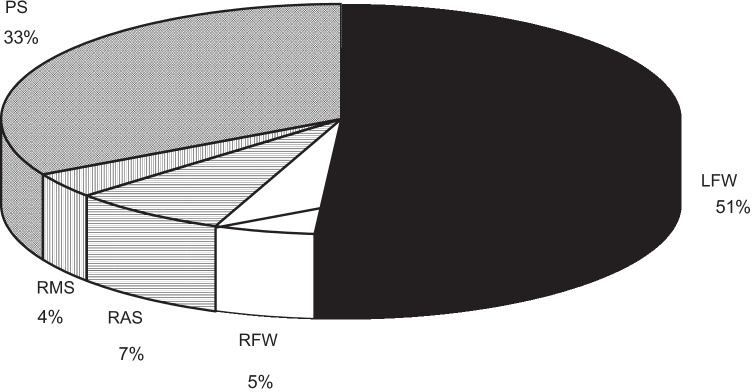
Distribution of accessory pathways. LFW: left free wall; RFW: right free wall; RAS: right anteroseptal; RMS: right midseptal; PS: posteroseptal

The distribution of pathways is shown in Fig. 2. APERP was recorded in 1069 patients, 425 women, and 644 men. The mean APERP was slightly shorter in non-septal pathways (299 ± 64 ms) compared to septal pathways (310 ± 74 ms) (*P* < 0.01). Mean APERP was similar in males (303 ± 72 ms) and females (305 ± 63 ms). There was no significant difference in the proportions with APERP ≤ 250 ms between men and women (139/644, 22% vs. 74/425, 17%) (*P* = 0.12) or between septal and non-septal pathways (*P* 1 = 0.29).

Arrhythmia was induced in 1036 (56%) patients. In all, 880/1036 (85%) had orthodromic tachycardia and 80/1036 (8%) had antidromic tachycardia. The antidromic tachycardias had shorter TCL than the orthodromic (320 ± 45 vs. 353 ± 60 ms) (*P* = 0.008). AF was induced in 170/1036 (17%) patients and it was preexcited in 125 (in 74% of cases with inducible AF). The shortest RR during AF was measured in 42 patients with a mean of 260 ms (range 160–400).

Ablation was attempted in 1702 (92%) patients. The primary success rate was 97.6%. The rate of major complications was 0.6% during the procedure and 0.8% at 30 days. The complication rate was markedly reduced during the last 5 years of the inclusion period — only 0.4% (two tamponades out of 459 procedures).

### Comparisons of the symptomatic and asymptomatic groups

**Fig. 3:**
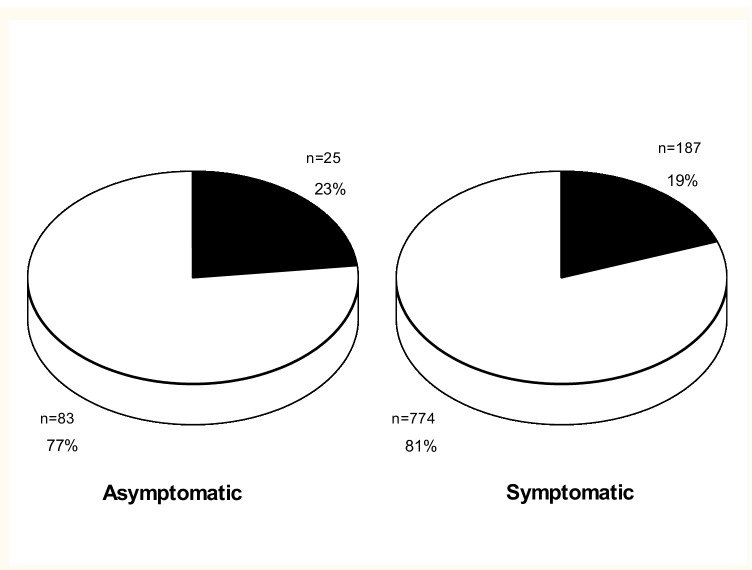
Proportion of pathways with accessory pathway effective refractory period, APERP, ≤ 250 ms (black) and pathways with APERP > 250 ms (white) in asymptomatic and symptomatic patients (*P* = 0.4)

**Fig. 4:**
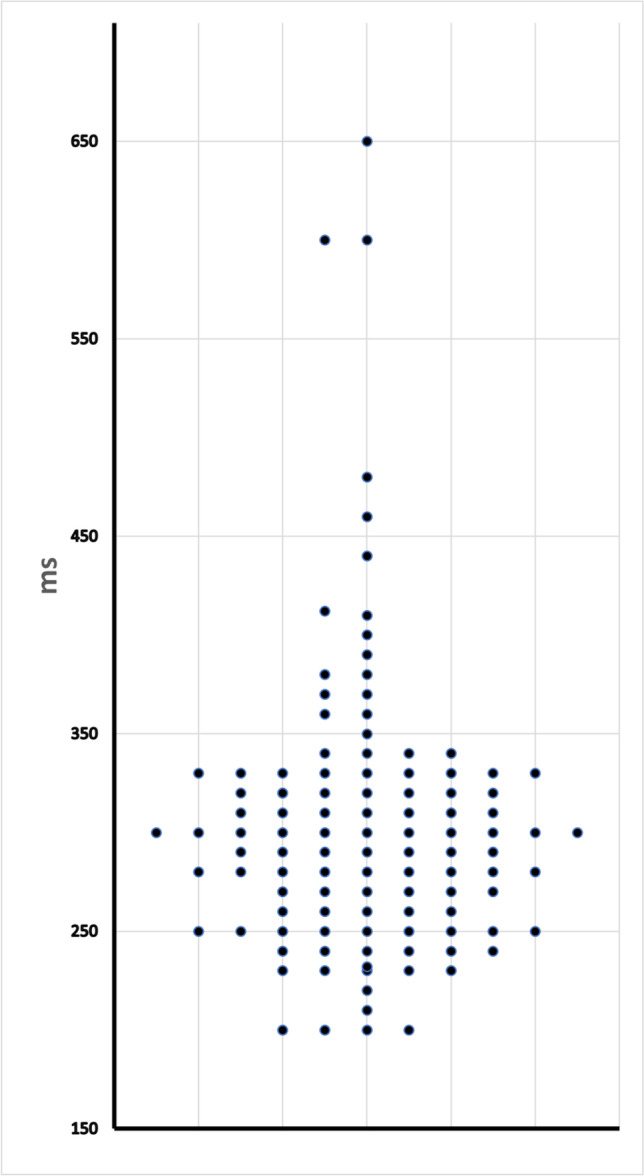
APERP in asymptomatic patients

The average age was slightly higher in the symptomatic compared to the asymptomatic group (36 ± 17 years vs. 33 ± 13 years) (*P* = 0.02). Mean APERP was similar in the symptomatic and asymptomatic groups (303 ± 68 ms and 307 ± 75 ms, respectively) (*P* = 0.6) as was the proportion of pathways with APERP ≤ 250 (187/949, 20% vs. 25/108, 23%) (*P* = 0.40). The proportion of patients with inducible arrhythmia was, however, larger in the symptomatic group (64 vs. 31%) (*P* < 0.001) (Figs. 3 and 4).

Five patients (all male; all left-sided APs) had suffered cardiac arrest before the EPS. This was the first symptom in one patient, and four patients had recurring palpitations and syncope before the cardiac arrest. VF was documented in four patients. In three of these patients, APERP was tested: 220 ms, 240 ms, and 260 ms. Arrhythmia was induced in three patients.

## Discussion

The main findings of this two-centre observational study of 1853 patients with preexcitation, 7–8% of them asymptomatic, were that the proportion of potentially dangerous APs and the mean APERP were similar in symptomatic and asymptomatic patients. Although arrhythmia was induced in twice as many symptomatic as asymptomatic patients, arrhythmia was nevertheless induced in 31% of asymptomatic patients, suggesting that a large proportion of them either may have asymptomatic arrhythmia episodes or are prone to manifest clinical tachycardia in the future, where the first symptom might be cardiac arrest.

An AP is considered to have a short refractory period and carry an increased risk for sudden cardiac death if one of three criteria is met: antegrade 1:1 conduction down to 250 ms during incremental atrial pacing; APERP ≤ 250 ms; or the shortest R-R interval during atrial fibrillation ≤ 250 ms. However, the optimal cut-off for a short and potentially dangerous refractory period is debatable and a range between 220 and 270 ms has been proposed [3–5, 8, 12]. In our study, 23% of asymptomatic patients had APERP ≤ 250 ms and 31% of them had inducible tachycardias. These findings agree with prior smaller studies that report that approximately 20% of asymptomatic patients demonstrate a rapid ventricular rate during AF induced during EPS [13–15]. The results suggest that asymptomatic preexcitation may be associated with short APERP and that invasive EPS could be considered selectively if non-invasive testing (ECG, exercise testing, and ambulatory ECG monitoring) is unhelpful (class IIa). Presently, there is an overall class IIa recommendation for EPS in individuals with asymptomatic preexcitation [9]. Our results definitely corroborate this recommendation and even provide arguments for a stronger recommendation, since a randomized trial would be problematic for ethical reasons.

We have the strategy to offer invasive EPS in patients with asymptomatic preexcitation referred to our centres. Our concern has been and still is that even asymptomatic preexcitation might be dangerous and pose a risk for sudden cardiac death, which is very difficult to estimate. Furthermore, an ablation is successful in ≥ 95% of cases, and there is a risk for major complications in considerably less than 1% of ablation attempts applying radiofrequency; however, the risk related to an invasive EPS alone is far less than 1% [16]. If an invasive risk assessment shows low probability for a life-threatening event and the AP is located mid-septal close to the AV-node, the procedure can always be stopped or an attempt to cryo-ablation be made. The results of this study provide support to continue with this strategy.

## Limitations

This retrospective study used data collected over a long period during which indications for invasive EPS have changed in our institutions, although not for the last 15 years. The APERP, a dynamic variable affected by autonomic tone and adrenergic drugs, was determined under basal conditions. Therefore, the proportion of potentially dangerous pathways could be underestimated. Presently, we test AP properties during isoprenaline infusion as part of the protocol when assessment during basal conditions does not show potentially dangerous AP properties [17].

## Conclusion

The current study reports on the common finding of a short APERP in asymptomatic and symptomatic patients with pre-excitation which highlights the limitations of the currently utilized invasive parameters for predicting rare potentially life-threatening arrhythmias. The results of this study support the present guideline recommendation (IIa) to carefully consider invasive risk assessment in select patients with asymptomatic pre-excitation.

## Data Availability

Not applicable.
